# Spectroscopic Analyses and Antimicrobial Activity of Novel Ciprofloxacin and 7-Hydroxy-4-methylcoumarin, the Plant-Based Natural Benzopyrone Derivative

**DOI:** 10.3390/ijms23148019

**Published:** 2022-07-20

**Authors:** Mohamed S. El-Attar, Sadeek A. Sadeek, Sherif M. Abd El-Hamid, Hazem S. Elshafie

**Affiliations:** 1 Department of Chemistry, Faculty of Science, Zagazig University, Zagazig 44519, Egypt; mselattar@zu.edu.eg (M.S.E.-A.); s_sadeek@zu.edu.eg (S.A.S.); 2 Department of Basic Science, Higher Future Institute of Engineering and Technology, Mansoura 35511, Egypt; sherifmohamed226@gmail.com; 3 School of Agricultural, Forestry, Food and Environmental Sciences, University of Basilicata, 85100 Potenza, Italy

**Keywords:** natural products, plant metabolites, semisynthetic bio-drugs, antimicrobial activity, coumarin, metal complexes, phytopathogens, human pathogens

## Abstract

Coumarin is highly distributed in nature, notably in higher plants. The biological features of coumarin include antibacterial, anticancer and antioxidant effects. It is well known that metal ions present in complexes accelerate the drug action and the efficacy of organic therapeutic agents. The main aim of the current study is the synthesis of different complexes of the interaction between ciprofloxacin hydrochloride (CIP) and coumarin derivative 7-hydroxy-4-methylcoumarin (HMC) with Zr(IV). The chelates of CIP with Zr(IV) were prepared and characterized by elemental analysis, melting point, conductance measurements, spectroscopic techniques involving IR, UV-Vis, ^1^H NMR, and thermal behavior (TG-DTG) in the presence of HMC, dimethylformamide (DMF), pyridine (Py), and triethylamine (Et_3_N). Results of molar conductivity tests showed that the new synthesized complexes are electrolytes with a 1:1 or 1:2 electrolyte ratio, with the chloride ions functioning as counter ions. According to IR spectra, CIP acts as a neutral bidentate ligand with Zr(IV) through one carboxylato oxygen and the carbonyl group, HMC as a monodentate through the carbonyl group, and DMF through the oxygen atom of the carbonyl group and the N atom of Py and Et_3_N. The thermal behavior of the complexes was carefully investigated using TG and DTG techniques. TG findings signal that water molecules are found as hydrated and coordinated. The thermal decomposition mechanisms proposed for CIP, HMC, and Zr(IV) complexes are discussed and the activation energies (E_a_), Gibbs free energies (∆G*), entropies (∆S*), and enthalpies (∆H*) of thermal decomposition reactions have been calculated using Coats–Redfern (CR) and Horowitz–Metzeger (HM) methods. The studied complexes were tested against some human pathogens and phytopathogens, including three Gram-positive bacteria (*Bacillus subtilis*, *B. cereus*, *Brevibacterium otitidis*) and three Gram-negative bacteria (*Escherichia* *coli*, *Pseudomonas aeruginosa* and *Klebsiella pneumoniae*), and compared to the free CIP and HMC parent compounds.

## 1. Introduction

Natural products are chemical compounds isolated from living organisms (plants, animals, fungi, and bacteria) [1,2]. In fact, the natural products of plant-based origin or their semisynthetic derivatives are the richest sources of biologically active compounds which have several benefits being utilized in different fields, such as nutrition, cosmetics, the medical/pharmaceutical field, and the agricultural and industrial fields [3,4].

Natural coumarin (2H-1-benzopyran-2-one) has very low antibacterial activity, but compounds having long chain hydrocarbon or carboxylic acids substitution such as ammoresinol, ostruthin, felamidin, and aegelinol, showed significant antibacterial activity against clinically isolated G+ve and G-ve bacterial strains [5,6,7]. Coumarin is highly distributed, generally, in nature and particularly in high plants [8]. The biological properties of different coumarins are well known, and they include anticoagulant, antiproliferative, antimicrobial, spasmolytic, antitumor, and antioxidant activities, among others [9,10,11]. Additionally, coumarin derivatives can yield a wide variety of metal complexes with different coordination modes, spectroscopic properties, and potential applications [10,11]. It is well known that metal ions present in complexes accelerate the drug action and the efficacy of organic therapeutic agents [12].

**Scheme 1:**
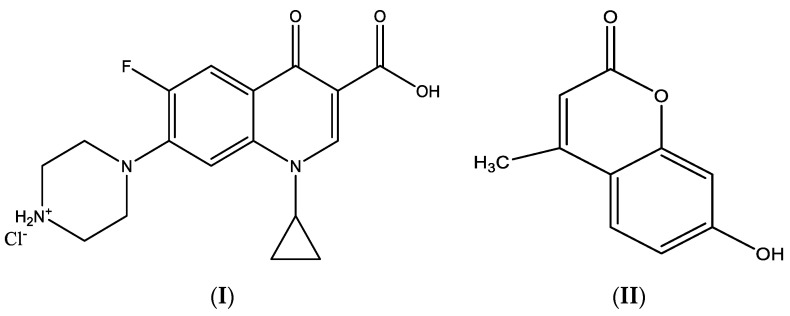
Structure of ciprofloxacin hydrochloride (CIP) (**I**) and 7-hydroxy-4-methylcoumarin (HMC) (**II**).

Quinolones are a group of synthetic antibacterial agents now in clinical use for over thirty years [13,14]. There are several reports about the proposed mechanism of the interaction between fluoroquinolones and metal cations, which have indicated chelation formed between the metal ions and the 4-oxo and the adjacent carboxyl group [15,16,17,18,19,20,21] or piperazine nitrogen atom [22,23,24]. Ciprofloxacin hydrochloride (CIP) (Scheme 1-I) is a second-generation fluoroquinolone that was synthesized for the first time in [25]. A well-known antibacterial drug with a wide spectrum of activity, it is extremely useful for the treatment of a variety of infections, such as Gram-positive (G+ve) and Gram-negative (G-ve) bacteria. It functions by inhibiting DNA gyrase, a type II topoisomerase, and topoisomerase IV, enzymes necessary to separate bacterial DNA, thereby inhibiting cell division [26,27]. CIP chelates with the metal ions as a bidentate ligand through the pyridone oxygen and one carboxylate oxygen [22,28,29,30]. Zr(IV) reacted with sparfloxacin, norfloxacin, moxifloxacin, lomefloxacin, ciprofloxacin, ofloxacin, carbamazebine, ibuprofen, meloxicam, and some tetradentate Schiff-base ligands, forming stable octahedral complexes [15,21,28,31,32,33,34,35,36,37,38,39]. The complexes exhibit highly significant antibacterial activity for G+ve and G-ve bacteria.

To continue our investigation in the field of fluoroquinolone complexes [15,31,32,40,41,42,43,44], we report in the present work the synthesis and characterization of new Zr(IV) complexes formed from the interaction of ciprofloxacin hydrochloride (CIP) and a coumarin derivative, 7-hydroxy-4-methylcoumarin (HMC) (Scheme 1-II), with Zr(IV) in the presence of DMF, Py, and Et_3_N in ethanol as a solvent and study the effect of change of solvates on the biological activity of CIP. The isolated solid complexes were characterized using spectroscopic and thermal analysis techniques. In addition, the antibacterial activity of the tested ligands and their complexes was tested against a variety of G+ve and G-ve bacteria.

## 2. Results and Discussion

The Zr(IV) ciprofloxacin and coumarin complexes were characterized and the data of elemental analysis of all complexes are very close to the theoretical values as listed in Table 1. The data in Table 1 show that the CIP:HMC:Zr(IV):L ratio is 1:1:1:1 and the compositions of the Zr(IV) complexes are [ZrO(CIP)(HMC)(H_2_O)Cl]Cl·5H_2_O and [ZrO(CIP)(HMC)(H_2_O)L]Cl_2_·nH_2_O (L = DMF, Py and Et_3_N and n = 10, 10 and 3, respectively). The new Zr(IV) complexes are stable at room temperature according to the thermogravimetric analysis. The IR spectroscopic and thermogravimetric data also confirm water in the composition of the complexes. Conductance data showed that all the complexes are electrolytes, indicating the chloride ions are located outside the coordination sphere [15,45]. Qualitative reactions revealed the presence of chloride as counter ions (the complex solutions give a white precipitate with AgNO_3_ solution) [46].

### 2.1. IR Absorption Spectra

The infrared spectra of the two ligands (CIP and HMC) and their complexes are shown in Figure S1, and their band assignments are listed in Table 2. IR spectra of all Zr(IV) complexes were compared with those of the two free ligands in order to determine the coordination sites that may be involved in the chelation mode. There were some guide peaks in ligand spectra, which are of good help for achieving this goal. The new peaks, position, and/or the intensities of these peaks were expected to be changed upon chelation, as were the guide peaks as well as the water of crystallization.

The IR spectrum of CIP ligand shows very strong bands at 1706 and 1620 cm^−1^ assignable to the stretching vibration of carboxylic *ν*(COOH) and the carbonyl group *ν*(C=O), respectively [31,32,40,41,42,47]. The absence of the first band in CIP and the shift of the second band of *ν*(C=O) to a lower value from 1620 cm^−1^ to an average value of 1522 cm^−1^ in Zr(IV) complexes reveals to the coordination of CIP through one O atom of both the carboxylato group and of the carbonyl group [31,32,40,41,42,43,44]. The asymmetric stretching vibration (*ν*_as_) of the ligated COO^−^ group is found in the range 1686–1628 cm^−1^ and the symmetric stretching vibration (*ν*_s_) of all complexes appear at ≈1389 cm^−1^ (Table 2). These data indicated that CIP ligated as monodetate through the oxygen atom of the COO^−^ group [44,48]. Additionally, the IR spectrum of the HMC ligand shows a very strong band at 1674 cm^−1^ assignable to the stretching vibration of the cyclic ester *ν*(C=O); this band is shifted to lower values (Table 2), indicating the coordination of HMC through the oxygen atom of the carbonyl group [25,26,27,28]. The presence of the broad bands in the range 3489–3411 cm^−1^ confirms the presence of H_2_O in all complexes [48]. A group of weak and medium intensity bands in the range 2928–2480 cm^−1^, which are assigned to *ν*(N-H) vibration of ^+^NH_2_ quaternized nitrogen of the piperazinyl group, indicates that the zwitterionic form of Gat-o-phdn is involved in the chelation to the metal ions investigated [33,34].

**Scheme 2:**
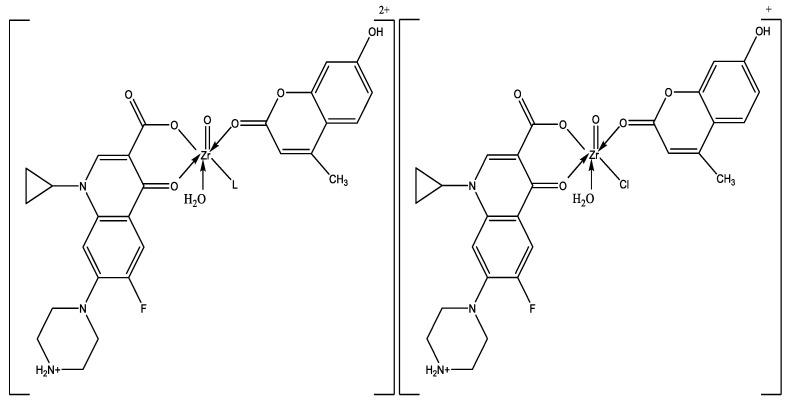
Coordination mode of Zr(ΙV) with CIP, HMC, and (L = DMF, Py, and Et_3_N).

The *ν*(Zr=O) in all complexes occurs in the range 845–849 cm^−1^ [33]. Some new bands with different intensities were observed at 625 and 540 cm^−1^ for **(A)** complex, at 637 and 556 cm^−1^ for **(B)** complex, at 679 and 539 cm^−1^ for **(C)** complex, and at 629 and 539 cm^−1^ for **(D)** complex, which are assigned to *ν* (Zr-O). According to the above data, the proposed structure formulas on the basis of the results discussed according to the infrared spectra are located as follows (Scheme 2).

### 2.2. Electronic Reflection Spectra

#### 2.2.1. UV-Vis Spectra

The Zr(IV) complexes were also confirmed by UV-Vis. spectra. Figure S2 gives the electronic spectra of CIP, HMC, and their Zr(IV) complexes in the range between 200 and 800 nm. CIP and HMC reflected at different distinct reflection bands (Table 3). For CIP, the bands at 243, 298, and 338 nm are attributed to π–π* and n–π* intra-ligand transitions (these transitions occur in case of unsaturated hydrocarbons which contain ketone groups) [34,49], while for HMC, the bands at 258 and 410 nm are attributed to π–π* and n–π* transitions, respectively. The absence of the reflection band at 243 nm in all four Zr(IV) complexes and the shift of the other bands to higher or lower values are attributed to complex formation between two ligands and Zr(IV). The new bands that appear in the range 514 to 572 nm are attributed to the ligand to Zr(IV) charge transfer [35,36,37,50].

#### 2.2.2. ^1^H NMR Spectra

The ^1^H NMR spectra were carried out to provide us information on the structures of all complexes. Figure S3 represents the ^1^H NMR spectra of CIP, HMC, **(A)**, **(B)**, **(C)**, and **(D)** compounds, which were carried out in DMSO-*d*_6_ solvent, and the data are listed in Table 4. The spectrum of the CIP ligand reveals a singlet signal at δ 11.00 ppm, assignable to the COOH proton [16], and the signal at δ 11.00 ppm disappeared in all complexes attributed to coordination of CIP to Zr(IV) through the carboxylate group [36,37,38,40]. The characteristic signals for quaternary nitrogen (–^+^NH_2_) showed at δ 2.11–2.35, 2.36, 2.12–2.36, and 2.36 ppm for the Zr(IV) complexes (Table 4); these data indicate CIP is neutral and present in zwitterionic state, which accords quite well with the practical data for molar conductivity and IR spectra [34]. The proton signal observed in the range δ 3.01–3.86 ppm, which may be assigned to the presence of water molecules, is in agreement with the suggested formulae of the Zr(IV) chelates [32,42]. Additionally, most of the signals of the free ligands were present in the spectra of the Zr(IV) complexes with chemical shift values depending on the binding to the Zr(IV) [24,51].

### 2.3. Thermal Studies

Thermal analyses (TG-DTG) were carried out for all compounds under N_2_ flow from ambient temperature to 800 °C (Figure S4) to establish the proposed formulae for the new complexes and also to decide whether the H_2_O molecules inside or outside the coordination sphere suggest a general scheme for the thermal decomposition of these chelates. The data of the TG and DTG curves of the compounds are listed in Table 5. The TG-DTG analyses of CIP were studied before [28,34]. Decomposition of HMC started at 50 °C and finished at 600 °C, with two stages. The first one occurred at maximum 60 °C with weight loss of 13.25% (calc. = 13.30%) due to the loss of 1.5 H_2_O. The second step found two maxima, 267 and 463 °C, with weight loss of 86.59% (calc. = 86.70%) assigned to the loss of 4C_2_H_2_+CO+CO_2_.

Thermal decomposition of the four complexes **(A)**, **(B)**, **(C)**, and **(D)** exhibit three main degradation steps. The first step of decomposition occurred at 100, 65, 60, and 50 °C, respectively, with weight losses of 11.27, 18.78, 18.69 and 6.26%, corresponding to the loss of 5, 10, 10, and 3 water molecules, respectively, in agreement with the theoretical values (11.35, 18.82, 18.71, and 6.29%, respectively). The second step occurred at one maximum temperature 324, 280, 210, and 183 °C, respectively, with weight losses of 33.35, 35.29, 35.70 and 42.60%, respectively, corresponding to the losses of 4C_2_H_2_+2CO_2_+2HCl, 2C_2_H_2_+2C_2_H_4_+5CO+NH_3_+2HCl, 4C_2_H_2_+C_2_H_4_+4CO+HCN+2HCl, and 2C_2_H_2_+4C_2_H_4_+4CO+NH_3_+2HCl. The final stage, found at a maximum temperature of 431 and 547 °C for complex **(A)** and at 493, 497, and 495 °C for the **(B)**, **(C)**, and **(D)** complexes, with weight losses 38.15, 32.89, 27.69, and 36.69%, respectively, with losses of 7C_2_H_2_+6CO+NH_3_+N_2_+HF, 7C_2_H_2_+C_2_N_2_+NH_3_+HF+CO_2_, 5C_2_H_2_+C_2_H_4_+CO_2_+NH_3_+HF+N_2_, and 7C_2_H_2_+C_2_N_2_+ NH_3_+HF+CO_2_, leaving ZrO_2_+C, ZrO_2_, ZrO_2_+4C, and ZrO_2_ for complexes **(A)**, **(B)**, **(C)**, and **(D)** respectively.

### 2.4. Thermodynamic Parameters

In order to assess the influences of the structural properties of the chelating agent, the order (n) and the heat of activation E_a_ of the various decomposition stages and the other thermodynamic parameters of enthalpies (ΔH*), entropies (ΔS*), and Gibbs free energies (ΔG*) were determined from the TG and DTG curves using the Coats–Redfern [52] and Horowitz–Metzger equations [53].

(1) $$\ln X\;=\;\ln\left[\frac{1-{\left(1-\propto \right)}^{1-n}}{T^{2}\left(1-n\right)}\right]=\;\ln\left(\frac{\mathrm{AR}}{\beta E}\right)-\frac{E_{a}}{\mathrm{RT}}\;\;\;\mathrm{for}\;n\neq 1$$

(2) $$\ln X\;=\;\ln\left[\frac{-\ln\left(1-\propto \right)}{T^{2}}\right]=\;\ln\left(\frac{\mathrm{AR}}{\beta E}\right)-\frac{E_{a}}{\mathrm{RT}}\;\;\;\mathrm{for}\;n\;=1$$

(3) $$\ln\left[-\ln\left(1-\propto \right)\right]=\frac{E_{a}\theta }{{\mathrm{RT}}_{s}^{2}}\;\;\;\mathrm{for}\;n\;=1$$

(4) $$\ln\left[\frac{1-{\left(1-\propto \right)}^{1-n}}{1-n}\right]=\;\ln\left(\frac{A}{\beta }\frac{{\mathrm{RT}}_{s}^{2}}{E}\right)-\frac{E_{a}}{{\mathrm{RT}}_{s}}+\frac{E_{a\;}\theta }{{\mathrm{RT}}_{s}^{2}}\;\;\;\mathrm{for}\;n\neq 1$$

(5) $${\Delta H}^{*}={\;E}_{a}-\mathrm{RT}\;$$

(6) $${\Delta S}^{*}=\;R\;\ln\frac{\mathrm{hA}}{K_{B}T}$$

(7) $${\Delta G}^{*}={\;\Delta H}^{*}-{T\Delta S}^{*}\;$$

The linearization curves are shown in Figure S5, and the kinetic parameters are summarized in Table 6. The correlation coefficients of the Arrhenius plots of the thermal decomposition steps were found to lie in the range 0.980–0.999, showing a good fit with linear function. These results show that all decomposition steps show a best fit for n = 1. The activation energies (E_a_) of decomposition were found to be in the range of 45.13–177.80 kJ mol^−1^, with negative value of ΔS* of some decomposition steps, indicating that the activated fragments have a more ordered structure than the undecomposed ones, and the later ones are slower than is normal [54]. The positive sign of ΔH* indicates that the decomposition stages are endothermic processes. The positive sign of ΔG* reveals that the free energy of the final residue is higher than that of the initial compound, and hence, all the decomposition steps are non-spontaneous processes.

### 2.5. Antibacterial Investigation

The susceptibility of certain bacterial strains to the ligands and their complexes was evaluated by measuring the diameter of inhibition zone (D.iz) in mm. Antibacterial activities of CIP, HMC, and their complexes were carried out with three G+ve strains: *Bacillus subtilis*, *Brevibacterium otitidis*, and *B. cereus* (G+ve) and *Escherichia coli*, *Pseudomonas aeruginosa*, and *Klebsiella pneumoniae* (G-ve). The tested solutions were prepared in DMSO-*d*_6_ and the results are presented in Table 7.

The synthesized compounds were found to have remarkable bactericidal effects against all tested bacterial strains. Figure S6 illustrates the statistical representation for biological activity of CIP, HMC, and their Zr(IV) complexes. The obtained results revealed that the complexes **(A)** and **(B)** showed very high significance against *B. subtilis*, with a high activity index, whereas complexes **(C)** and **(D)** showed highly significant activity against *B. subtilis*, more than CIP, HMC, and standard antibiotic control. All Zr(IV) complexes were less efficient against *Br. otitidis* (except complex **(A)**) and *B. cereus* than CIP and standard antibiotic control (Table 7). For G-ve bacteria strains, the complex **(D)** showed highly significant activity against *E. coli* and *P. aeruginosa* more than CIP, HMC, and standard antibiotic control. The complex **(C)** showed highly significant activity against *P. aeruginosa*, and complex **(B)** showed significant activity against *P. aeruginosa* and *K. pneumoniae*; complex **(A)** showed significant activity against *E. coli* more than CIP, HMC and standard antibiotic control (Table 7). Even if HMC showed a moderate antimicrobial effect against the tested bacterial strains, these results are promising regarding the use of natural substances in semisynthetic chelates and compared to parent ligands.

In particular, the lowest MIC for *E. coli* was measured in the cases of complex **(B)** and HMC ligand, at 0.50 μg/mL, followed by complex **(C)** and CIP ligand at 0.75 μg/mL, whereas complexes **(A)** and **(D)** showed the highest MIC values at 1.00 μg/mL. The MIC for *P*. *aeruginosa* was 0.25 μg/mL for HMC and complex **(B)**, followed by CIP ligand at 0.50 μg/mL, complex **(C)** at 0.75 μg/mL, whereas complexes **(A)** and **(D)** showed the highest MIC values at 1.00 μg/mL. Additionally, the lowest MIC value for *K. pneumoniae* was shown by HMC ligand at 0.25 μg/mL, followed by complex **(D)** at 0.50 μg/mL, then complex **(C)** and CIP ligand at 0.75 μg/mL, followed by complexes **(A)** and **(B)** at 1.00 μg/mL. The MIC for *B. subtilis*, *B. cereus*, and *Br. otitidis* was recorded at 0.25 μg/mL for HMC. However, only CIP recorded MIC values against *B. subtilis* and *B. cereus* at 0.50 μg/mL and 0.75 μg/mL against *Br. Otitidis*. The MIC value for *B. subtilis* was recorded at 0.50 μg/mL for complexes **(C)** and **(D)** and at 0.75 μg/mL for complexes **(A)** and **(B)**. For *B. cereus*, the MIC values were recorded at 0.25 μg/mL for complex **(B)**, at 0.50 μg/mL for complex **(C)**, and at 0.75 μg/mL for complexes **(A)** and **(D)**. Finally, for *Br. otitidis*, the MIC values were found at 0.25 μg/mL corresponding to complex **(D)**, at 0.50 μg/mL for complexes **(A)** and **(B)**, and at 0.75 μg/mL for complex **(C)**.

The chelation process increased the potency of the coumarin derivatives and ciprofloxacin as a bacteriostatic agent [12,55,56]. Chelation considerably reduced the polarity of the metal ion because of the partial sharing of its positive charge with the donor groups and possible p-electron delocalization over the chelate ring. On the other hand, such chelation increased the lipophilic properties of the central metal ion, which subsequently favored the permeation through the lipid layer of the cell membrane [56]. Thus, the increased lipophilicity can enhance the penetration of the complexes into the lipid membranes and block the metal binding sites in the enzymes of microorganisms [57,58].

## 3. Materials and Methods

### 3.1. Chemicals

CIP was obtained from the Egyptian International Pharmaceutical Industrial Company (EIPICO), ethanol, AgNO_3_, Py, DMF, Et_3_N, ZrOCl_2_·8H_2_O, potassium dichromate, concentrated sulfuric acid, commercial grade concentrated nitric acid 69%, hydrogen peroxide 20%, gallein (Pyrogallolphthalein), silver nitrate, resorcinol, and ethyl acetoacetate were provided by Sigma Aldrich Chemicals (Darmstadt, Germany) and Fluka Chemicals (Rodano, Italy).

### 3.2. Synthesis of Coumarin

7-hydroxy-4-methyl-coumarin (HMC) (Scheme 1-II) was synthesized in pure solid state by addition of 100 g (0.91 mol) of resorcinol in 130.5 mL (1.03 mol) of redistilled ethyl acetoacetate dropwise with stirring (below 10 °C), then keeping the mixture at room temperature for 18 h and then pouring it into a mixture of 2 kg of crushed ice with vigorous stirring and 3 L of water, then collecting the precipitate by suction filtration and washing it with three 25 mL portions of cold water. The yellow precipitate was filtered at the pump, washed with cold water and dried under vacuum over CaCl_2_ in desiccator and recrystallize from 95% ethanol [59].

### 3.3. Synthesis of Ciprofloxacin/Coumarin Zr(IV) Complexes

The faint brown solid complex [ZrO(CIP)(HMC)(H_2_O)Cl]Cl·5H_2_O **(A)** was prepared by adding 1 mmol (0.3223 g) of ZrOCl_2_·8H_2_O in 30 mL ethanol dropwise to a stirred mixture solution of 1 mmol (0.3678 g) CIP, 1 mmol (0.0400 g) of NaOH, and 1 mmol (0.2032 g) of HMC in 20 mL ethanol. The reaction mixture was stirred for 2 days at room temperature. The precipitate was filtered off and dried under vacuum over CaCl_2_.

The green solid complex [ZrO(CIP)(HMC)(DMF)(H_2_O)]Cl_2_·10H_2_O **(B)** was synthesized by adding 1 mmol (0.3223 g) of ZrOCl_2_·8H_2_O dropwise to a stirred mixture solution containing 1 mmol (0.3678 g) of CIP, 1 mmol (0.040 g) of NaOH, 1 mmol (0.2032 g) of HMC, and 1 mmol (3 mL) of DMF in 50 mL ethanol. The reaction mixture was stirred for 22h at room temperature. The precipitate was filtered off and dried under vacuum over CaCl_2_.

The greenish yellow and brownish yellow solid complexes of [ZrO(CIP)(HMC)(Py)(H_2_O)]Cl_2_·10H_2_O **(C)** and [ZrO(CIP)(HMC)(Et_3_N)(H_2_O)]Cl_2_·3H_2_O **(D)** were prepared in a similar manner, described above, using 1 mmol (0.3223 g) of ZrOCl_2_·8H_2_O with 1 mmol (2 mL) of Py, 1 mmol (3 mL) of Et_3_N, respectively, and using 40 mL ethanol as a solvent. These solid complexes were filtered off and dried under vacuum over CaCl_2_. After using slow evaporation and cooling crystallization procedures, monocrystals could not be formed for X-ray crystallography. The four new prepared complexes were characterized by elemental analysis, molar conductivity, IR, UV-Vis, ^1^H NMR, and thermal analyses.

### 3.4. Instruments

Elemental C, H, N, and halogen analyses were carried out on a Perkin-Elmer CHN 2400. The percentage of Zr(IV) was determined by three analytical methods, complexometric titration, thermogravimetry, and atomic absorption. Gravimetric determination was carried out by transforming the solid products into zirconium oxide and also determined by using atomic absorption method [15,28]. Spectrometer model PYE-UNICAM SP 1900 fitted with the corresponding lamp was used for this purposed. Infrared spectra of the prepared complexes were recorded as KBr discs on FTIR 460 PLUS in the range from 4000 to 400 cm^−1^. ^1^H NMR spectra for complexes were recorded on Varian Mercury VX-300 NMR Spectrometer using DMSO-*d*_6_ as solvent. Thermal analyses (TG-DTG) measurements were carried out in dynamic N_2_ atmosphere (20 mL min^−1^) with a heating rate of 10 °C/min using Shimadzu TGA-50H thermal analyzer within the temperature range from room temperature to 800 °C. UV-3101PC Shimadzu was used to obtain the electronic spectra for the prepared complexes. The solid reflection spectra were recorded with KBr discs. Magnetic properties were carried out on a Sherwood scientific magnetic balance using Gouy method, using Hg[Co(SCN)_4_] as calibrant. Molar conductivities of the solutions of the ligands and prepared complexes in DMF at 1 × 10^−3^ M were measured on CONSORT K410. All measurements were carried out at ambient temperature with freshly prepared solutions.

### 3.5. Antimicrobial Investigation and MIC Determination

Antibacterial activity of the ligands and their zirconium complexes was investigated by a previously reported modified method of Beecher and Wong [60] and Elshafie et al. [61] against different bacterial species, *B. subtilis*, *Br. otitidis*, and *B. cereus* (G+ve) and *E. coli*, *P. aeruginosa*, and *K. pneumonia*e (G-ve).

The tested microorganisms were isolated from Egyptian soil and identified according to the standard bacteriological keys for identification of bacteria as stock cultures in the microbiology laboratory, Faculty of Science, Zagazig University. The Muller–Hington agar (30.0% beef extract, 1.75% casein hydrolysate, 0.15% starch, and 1.7% agar) was prepared and then cooled and seeded with tested microorganisms. After solidification, 5 mm diameter holes were punched with a sterile cork-borer and 100 µL of each investigated compounds, i.e., ligands and their zirconium complexes, were introduced into holes after being dissolved in DMSO at 10^−4^ M. The culture plates were incubated at 37 °C for 20 h. The diameters of the inhibitory zones (D.iz) in (mm) were used to determine the antibacterial activity, whereas the bacterial growth inhibition was estimated using ciprofloxacin as a positive control. The following formula was used to compute the activity index for the produced compounds [39]:

$$\mathrm{Activity}\;\mathrm{index}\;(\%)=\frac{\mathrm{Zone}\;\mathrm{of}\;\mathrm{inhibition}\;\mathrm{by}\;\mathrm{test}\;\mathrm{compound}\;\left(\mathrm{diameter}\right)}{\mathrm{Zone}\;\mathrm{of}\;\mathrm{inhibition}\;\mathrm{by}\;\mathrm{standard}\;\left(\mathrm{diameter}\right)}\;\times \;100$$

On the other hand, the minimal inhibitory concentrations (MIC) for CIP, HMC, and their Zr(IV) complexes **(A)**, **(B)**, **(C)**, and **(D)** against the abovementioned tested bacterial strains was carried out following the standard broth microdilution method in LB broth [62]. The studied compounds were tested at concentrations ranging from 0.25 to 1.0 μg/mL, whereas the carrier solvent DMSO was used as a control.

## 4. Conclusions

Molecular structures of the four novel Zr(IV) complexes were supported employing diverse physicochemical techniques. According to the findings, CIP serves as bi-dentate ligands via pyridone oxygen and carboxylate oxygen for CIP and monodentate HMC, DMF, Py, and Et_3_N through the oxygen atom of the carbonyl group and the N atom, respectively. All complexes have a coordination number of six. The findings of this study confirm the postulated octahedral shape of the metal complexes and constitute a beneficial molecular arrangement. The kinetic parameters of the thermal decomposition phases were determined using the Coats–Redfern and Horowitz–Metzeger equations. The studied Zr(IV) complexes in different coordination modes demonstrated promising biological activity against some G+ve and G-ve bacterial strains, where the Zr(IV) can accelerate the drug action. The interesting biological activity of the studied complexes was due to the action of chelation process, which increased the potency of ciprofloxacin as a bacteriostatic agent. Complexes **(A)** and **(B)** showed the highest significant antimicrobial effect against *B. subtilis* with an MIC value at 0.75 μg/mL, whereas the complexes **(C)** and **(D)** showed highly significant activity, with a MIC value 0.50 μg/mL against *B. subtilis*, more than CIP, HMC, and standard antibiotic control. Further studies remain necessary to determine the efficient concentrations of the studied coumarin derivatives in the prepared complexes and their modes of action.

## Figures and Tables

**Table 1:** Elemental analysis and physico-analytical data for CIP, HMC, and their Zr(IV) complexes **(A)**, **(B)**, **(C)**, and **(D)**.

Compounds M.Wt. (M.F.)	Yield%	mp/°C	Color	Content (calc.) Found (%)					Λ (S cm^2^ mol^−1^)
				C	H	N	M	Cl	
**CIP** 367.45 (C_17_H_19_N_3_O_3_FCl)	-	305	White	(55.52) 55.48	(5.17) 5.10	(11.43) 11.34	-	(9.65) 9.56	70.20
**HMC** 203.00 (C_10_H_11_O_4.5_)	79	190	Yellow	(59.11) 59.03	(5.42) 5.36	- -	- -	- -	15.12
**(A)** 793.12 (C_27_H_38_N_3_O_13_FCl_2_Zr)	75	290	Faint brown	(40.85) 40.73	(4.79) 4.70	(5.30) 5.23	(11.50) 11.42	(8.94) 8.88	76.23
**(B)** 956.12 (C_30_H_55_N_4_O_19_FCl_2_Zr)	80	200	Green	(37.65) 37.56	(5.75) 5.69	(5.86) 5.76	(9.54) 9.45	(7.42) 7.35	122.15
**(C)** 962.12 (C_32_H_53_N_4_O_18_FCl_2_Zr)	78	340	Greenish yellow	(39.91) 39.81	(5.51) 5.45	(5.82) 5.73	(9.48) 9.40	(7.37) 7.29	125.26
**(D)** 858.12 (C_33_H_49_N_4_O_11_FCl_2_Zr)	85	160	Brownish yellow	(46.15) 46.02	(5.71) 5.63	(6.53) 6.45	(10.63) 10.55	(8.26) 8.17	120.30

**Table 2:** Selected infrared absorption frequencies (cm^−1^) for CIP, HMC, and their Zr(IV) complexes **(A)**, **(B)**, **(C)**, and **(D)**.

Compounds	*ν*(O-H); H_2_O; COOH	*ν*(C=O); COOH	*ν*_as_(COO^−^)	*ν*(C=O)	*ν*_s_(COO^−^)	*ν* (Zr = O)	*ν*(M-O) and *ν*(M-N)
**CIP**	3530 m	1706 vs	-	1620 vs	-	-	-
**HMC**	3499 ms	-	-	1674 vs	-	-	-
**(A)**	3489 w	-	1670 ms	1609 vs 1521 w	1389 vs	845 s	625 m, 540 m
**(B)**	3533 w 3411 w	-	1628 s,br	1539 w	1389 s	848 w	637 ms, 560 wbr
**(C)**	3444 w	-	1678 m	1624 s 1572 m	1389 vs	845 s	679 ms, 539 w
**(D)**	3517 mbr	-	1686 m	1620 mbr 1522 m	1389 vs	849 vs	629 s, 539 w

Keys: s = strong, w = weak, m = medium, br = broad, *ν* = stretching.

**Table 3:** UV–Vis spectra for CIP, HMC, and their Zr(IV) complexes **(A)**, **(B)**, **(C)**, and **(D)**.

Assignments (nm)	CIP	HMC	(A)	(B)	(C)	(D)
π–π* transitions	243, 298	258	252, 304	277, 312	282, 302	282, 302
n–π* transitions	338	410	385	404, 431	323, 407, 431	323, 407, 442
Ligand-metal charge transfer	-	-	525, 574	522, 545, 567	514, 572	514, 572

**Table 4:** ^1^H NMR values (ppm) and tentative assignments for CIP, HMC, and their Zr(IV) complexes **(A)**, **(B)**, **(C)**, and **(D)**.

Assignments (ppm)	CIP	HMC	(A)	(B)	(C)	(D)
δH, -CH and -CH_3_	1.33	-	0.96–1.35	1.19–1.31	0.79–1.33	1.13–1.24
δH, -NH; piperazine	2.00	-	-	-	-	-
δH, -^+^NH_2_	-	-	2.11–2.35	2.36	2.12–2.36	2.36
δH, -CH_2_ aliphatic	2.78, 3.46	2.35–2.50	2.41–2.51	2.50	2.45–2.56	2.49–2.51
δH, H_2_O	-	3.79	3.15–3.86	3.37, 3.57	3.04–3.63	3.01–3.36
δH, -CH_2_ aromatic	6.04–8.66	6.12–7.60	5.09–9.88	6.12–8.80	6.12–9.91	6.12–7.60
δH, -COOH and -OH	11.00	10.50	10.69	10.67	10.68	10.20

**Table 5:** The maximum temperature T_max_ (°C) and weight loss values of the decomposition stages for HMC and their Zr(IV) complexes **(A)**, **(B)**, **(C)**, and **(D)**.

Compounds	Decomposition	T_max_ (°C)	Weight Loss (%)		Lost Species
			Calc.	Found	
**HMC**	First step Second step Total loss	60 267, 463	13.30 86.70 100	13.25 86.59 99.84	1.5H_2_O 4C_2_H_2_+CO+CO_2_
**(A)**	First step Second step Third step Total loss, Residue	100 324 431, 547	11.35 33.40 38.20 82.95, 17.05	11.27 33.35 38.15 82.77, 17.23	5H_2_O 4C_2_H_2_+2CO_2_+2HCl 7C_2_H_2_+6CO+NH_3_+N_2_+HF ZrO_2_+C
**(B)**	First step Second step Third step Total loss, Residue	65 280 493	18.82 35.34 32.95 87.11, 12.89	18.78 35.29 32.89 86.96, 13.04	10H_2_O 2C_2_H_2_+2C_2_H_4_+5CO+NH_3_+2HCl 7C_2_H_2_+C_2_N_2_+NH_3_+HF+CO_2_ ZrO_2_
**(C)**	First step Second step Third step Total loss, Residue	60 210 497	18.71 35.74 27.75 82.20, 17.80	18.69 35.70 27.69 82.08, 17.92	10H_2_O 4C_2_H_2_+C_2_H_4_+4CO+HCN+2HCl 5C_2_H_2_+C_2_H_4_+CO_2_+NH_3_+HF+N_2_ ZrO_2_+4C
**(D)**	First step Second step Third step Total loss, Residue	50 183 495	6.29 42.64 36.71 85.64, 14.36	6.26 42.60 36.69 85.55, 14.45	3H_2_O 2C_2_H_2_+4C_2_H_4_+4CO+ NH_3_+2HCl 7C_2_H_2_+C_2_N_2_+ NH_3_+HF+CO_2_ ZrO_2_

**Table 6:** Thermal behavior and kinetic parameters determined using Coats–Redfern (CR) and Horowitz–Metzger (HM) equations, operated for HMC and their Zr(IV) complexes **(A)**, **(B)**, **(C)**, and **(D)**.

Compounds	Decomposition Range (K)	T_s_ (K)	Method	Parameter					R ^a^	SD ^b^
				E_a_ (kJ/mol^−1^)	A (s^−1^)	ΔS* (kJ/mol.K)	ΔH* (kJ/mol)	ΔG* (kJ/mol)		
**HMC**	313–393	333	CR HM	53.26 55.33	1.14 × 10^6^ 2.39 × 10^5^	−0.1297 −0.1156	50.52 52.59	93.21 90.64	0.999 0.998	0.03 0.81
	471–593	540	CR HM	68.67 85.48	1.27 × 10^5^ 8.04 × 10^6^	−0.1513 −0.1169	64.57 81.38	139.17 139.00	0.995 0.995	0.05 0.11
**(A)**	673–754	704	CR HM	106.80 115.20	1.8 × 10^8^ 2.5 × 10^9^	−0.092 −0.070	102.42 110.87	150.49 147.55	0.990 0.990	0.13 0.15
**(B)**	693–803	766	CR HM	61.43 67.39	1.21 × 10^4^ 1.08 × 10^5^	−0.1708 −0.1526	57.41 63.36	140.08 137.21	0.990 0.990	0.11 0.14
**(C)**	708–823	770	CR HM	177.80 193.31	5.96 × 10^10^ 7.91 × 10^11^	−0.046 −0.025	171.8 187.32	204.90 204.90	0.998 0.998	0.05 0.05
**(D)**	423–493	456	CR HM	45.13 57.53	5.4 × 10^2^ 8.3 × 10^3^	−0.217 −0.175	40.45 52.86	162.34 151.39	0.994 0.997	0.074 0.07
	743–818	768	CR HM	77.66 92.41	1.46 × 10^2^ 2.16 × 10^3^	−0.2119 −0.1895	70.85 85.60	244.39 240.77	0.990 0.980	0.15 0.20

^a^ = correlation coefficients of the Arrhenius plots and ^b^ = standard deviation.

**Table 7:** Antibacterial activity of CIP, HMC, and their Zr(IV) complexes **(A)**, **(B)**, **(C)**, and **(D)** against G+ve and G-ve bacterial strains.

Tested Compounds		Tested G+ve Bacterial Strains								
		*B. subtilis*			*B. cereus*			*Br. otitidis*		
		D.iz ^a^ (mm)	AI ^b^ (%)	MIC ^c^ (μg/mL)	D.iz (mm)	AI (%)	MIC (μg/mL)	D.iz (mm)	AI (%)	MIC (μg/mL)
**CIP**		26 ± 0.58	-	0.50 ± 0.005	34 ± 0.43	-	0.50 ± 0.007	22 ± 0.29	-	0.75 ± 0.005
**HMC**		18 ± 0.22	0.69	0.25 ± 0.03	8 ± 0.15	0.24	0.25 ± 0.01	7 ± 0.11	0.32	0.25 ± 0.006
**(A)**		66^+3^ ± 0.26	2.5	0.75 ± 0.01	27 ± 0.36	0.79	0.75 ± 0.006	27^+1^ ± 0.65	1.23	0.50 ± 0.01
**(B)**		61^+3^ ± 0.25	2.35	0.75 ± 0.006	24 ± 0.24	0.71	0.25 ± 0.02	19 ± 0.21	0.86	0.50 ± 0.03
**(C)**		58^+2^ ± 0.45	2.23	0.50 ± 0.007	26 ± 0.32	0.76	0.50 ± 0.01	19 ± 0.17	0.86	0.75 ± 0.007
**(D)**		49^+2^ ± 0.49	1.88	0.50 ± 0.01	22 ± 0.15	0.65	0.75 ± 0.03	16 ± 0.09	0.73	0.25 ± 0.01
ZrOCl_2_·8H_2_O		0	0	-	0	0	-	0	0	-
Control (DMF)		0	0	-	0	0	-	0	0	-
Standards	Moxifloxacin	40 ± 0.5	1.54	-	36 ± 1.2	1.06	-	25 ± 0.24	1.14	-
	Lomefloxacin	24 ± 0.2	0.92	-	25 ± 0.5	0.74	-	26 ± 0.31	1.18	-
**Tested Compounds**		**Tested G-ve Bacterial Strains**								
		***E. coli***			***P. aeruginosa***			***K. pneumoniae***		
		**D.iz ^a^** **(mm)**	**AI ^b^** **(%)**	**MIC ^c^ (μg/mL)**	**D.iz ** **(mm)**	**AI** **(%)**	**MIC (μg/mL)**	**D.iz** **(mm)**	**AI** **(%)**	**MIC (μg/mL)**
**CIP**		28 ± 0.3	-	0.75 ± 0.01	23 ± 0.3	-	0.50 ± 0.02	20 ± 0.4	-	0.75 ± 0.03
**HMC**		7 ± 0.05	0.25	0.50 ± 0.007	ND	-	0.25 ± 0.01	12 ± 0.31	0.60	0.25 ± 0.007
**(A)**		31^+1^ ± 0.81	1.1	1.0 ± 0.02	-	-	1.0 ± 0.007	16 ± 0.19	0.8	1.0 ± 0.02
**(B)**		26 ± 0.23	0.93	0.50 ± 0.005	29^+1^ ± 0.92	1.26	0.50 ± 0.005	27^+1^ ± 0.88	1.35	1.0 ± 0.01
**(C)**		28 ± 0.09	1	0.75 ± 0.03	33^+2^ ± 0.68	1.43	0.75 ± 0.01	21^NS^ ± 0.60	1.05	0.75 ± 0.005
**(D)**		35^+2^ ± 0.45	1.25	1.0 ± 0.006	34^+2^ ± 0.76	1.48	1.0 ± 0.03	21^NS^ ± 0.51	1.05	0.50 ± 0.02
ZrOCl_2_·8H_2_O		0	0	-	0	0	-	0	0	-
Control (DMF)		0	0	-	0	0	-	0	0	-
Standards	Moxifloxacin	22 ± 1.7	0.78	-	22 ± 0.3	0.96	-	16 ± 0.1	-	-
	Lomefloxacin	17 ± 0.1	0.61	-	13 ± 0.3	0.56	-	19 ± 0.1	-	-

Statistical significance: P^NS^, P not significant, *p* *> 0.05*; P^+1^, P significant, *p* *< 0.05*; P^+2^, P highly significant, *p* *< 0.01*; P^+3^, P very highly significant, *p* *< 0.001*; student’s *t-*test (paired). ^a^ D.iz: diameter of inhibition zone (mm); ^b^ AI: activity index (%); ^c^ MIC: minimum inhibitory concentration (μg/mL); ND: not detectable.

## Data Availability

Not applicable.

## Supplementary Materials

The following supporting information can be downloaded at: https://www.mdpi.com/article/10.3390/ijms23148019/s1.
