# Supplementary material for: Spectroscopic Analyses and Antimicrobial Activity of Novel Ciprofloxacin and 7-Hydroxy-4-methylcoumarin, the Plant-Based Natural Benzopyrone Derivative
Source: Int J Mol Sci. 2022 Jul 20;23(14):8019. doi: 10.3390/ijms23148019 (PMC9316271; doi:10.3390/ijms23148019)
Supplement: Supplementary file 1 [file ijms-23-08019-s001.zip › ijms-1808278-supplementary.pdf]

Spectroscopic Analyses and Antimicrobial Activity of Novel Ciprofloxacin and 7-Hydroxy-4-methylcoumarin, the Plant-Based Natural Benzopyrone Derivative

Mohamed S. El-Attar [1], Sadeek A. Sadeek [1], Sherif M. Abd El-Hamid [2] and Hazem S. Elshafie [3,*]

[1] Department of Chemistry, Faculty of Science, Zagazig University, Zagazig 44519, Egypt; mselattar@zu.edu.eg (M.S.E.-A.); s_sadeek@zu.edu.eg (S.A.S.)
[2] Department of Basic Science, Higher Future Institute of Engineering and Technology, Mansoura 35511, Egypt; sherifmohamed226@gmail.com
[3] School of Agricultural, Forestry, Food and Environmental Sciences, University of Basilicata, 85100 Potenza, Italy
* Correspondence: hazem.elshafie@unibas.it; Tel.: +39-0971-205522; Fax: +39-0971-205503

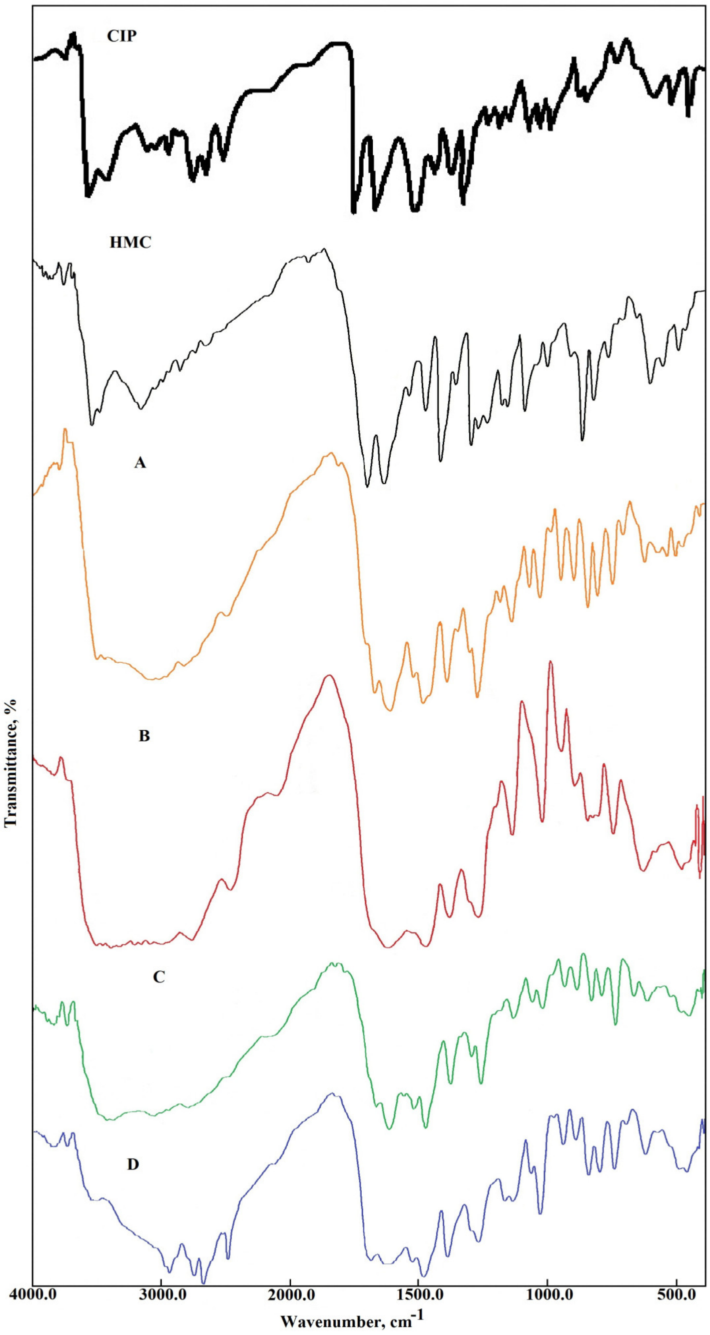

Figure S1. Infrared spectra for CIP, HMC and their Zr(IV) complexes (**A**) $[ZrO(CIP)(HMC)(H_2O)Cl]Cl.5H_2O$, **(B)** $[ZrO(CIP)(HMC)(DMF)(H_2O)]Cl_2.10H_2O$, **(C)** $[ZrO(CIP)(HMC)(Py)(H_2O)]Cl_2.10H_2O$ and **(D)** $[ZrO(CIP)(HMC)(Et_3N)(H_2O)]Cl_2.3H_2O$.

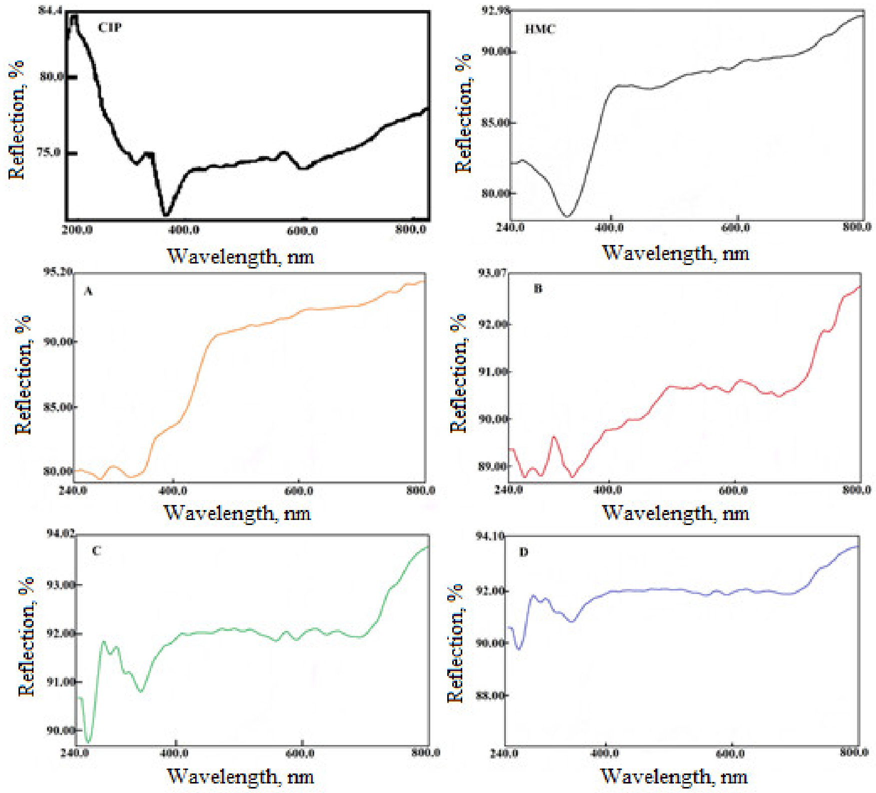

Figure S2. UV-Vis spectra for CIP, HMC and their Zr(IV) complexes (**A)** [ZrO(CIP)(HMC)(H_2O)Cl]Cl.5H_2O, **(B)** [ZrO(CIP)(HMC)(DMF)(H_2O)]Cl_2.10H_2O, **(C)** [ZrO(CIP)(HMC)(Py)(H_2O)]Cl_2.10H_2O and **(D)** [ZrO(CIP)(HMC)(Et_3N)(H_2O)]Cl_2.3H_2O.

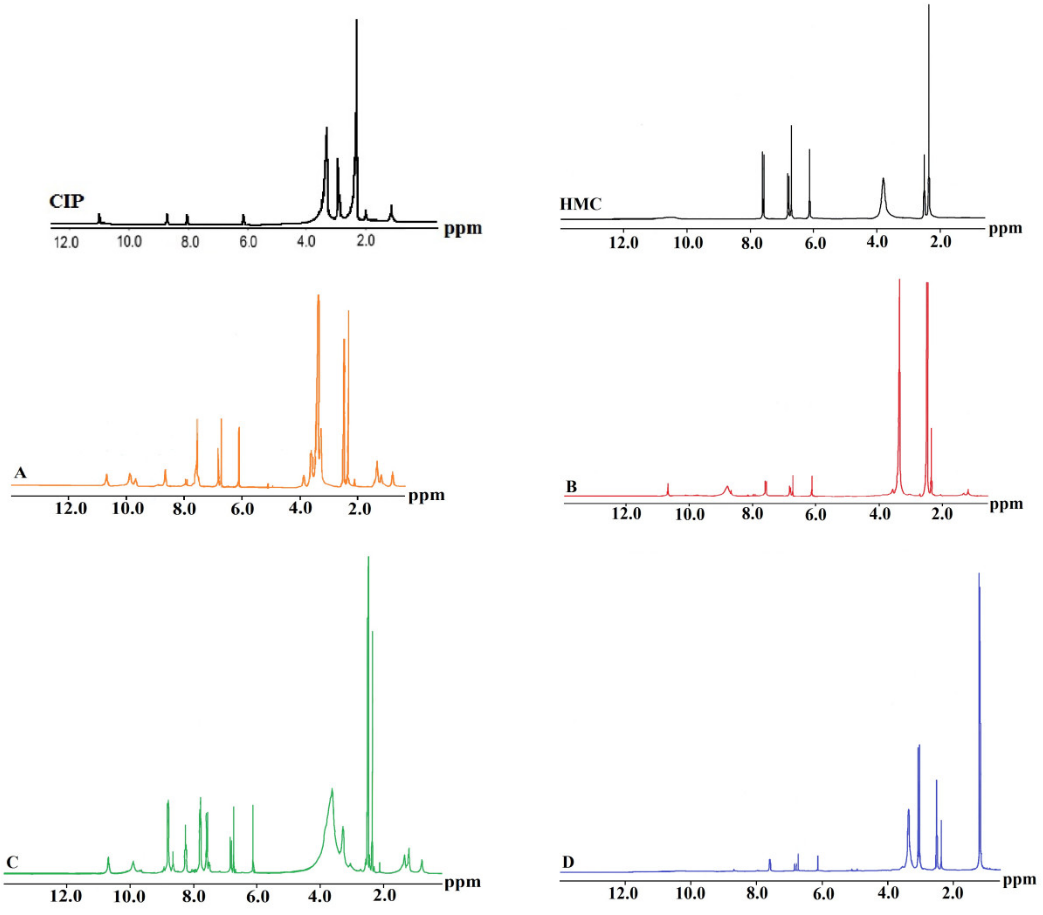

Figure S3. ^{1}H NMR spectra for CIP, HMC and their Zr(IV) complexes **(A)** [ZrO(CIP)(HMC)(H_2O)Cl]Cl.5H_2O, **(B)** [ZrO(CIP)(HMC)(DMF)(H_2O)]Cl_2.10H_2O, **(C)** [ZrO(CIP)(HMC)(Py)(H_2O)]Cl_2.10H_2O and **(D)** [ZrO(CIP)(HMC)(Et_3N)(H_2O)]Cl_2.3H_2O.

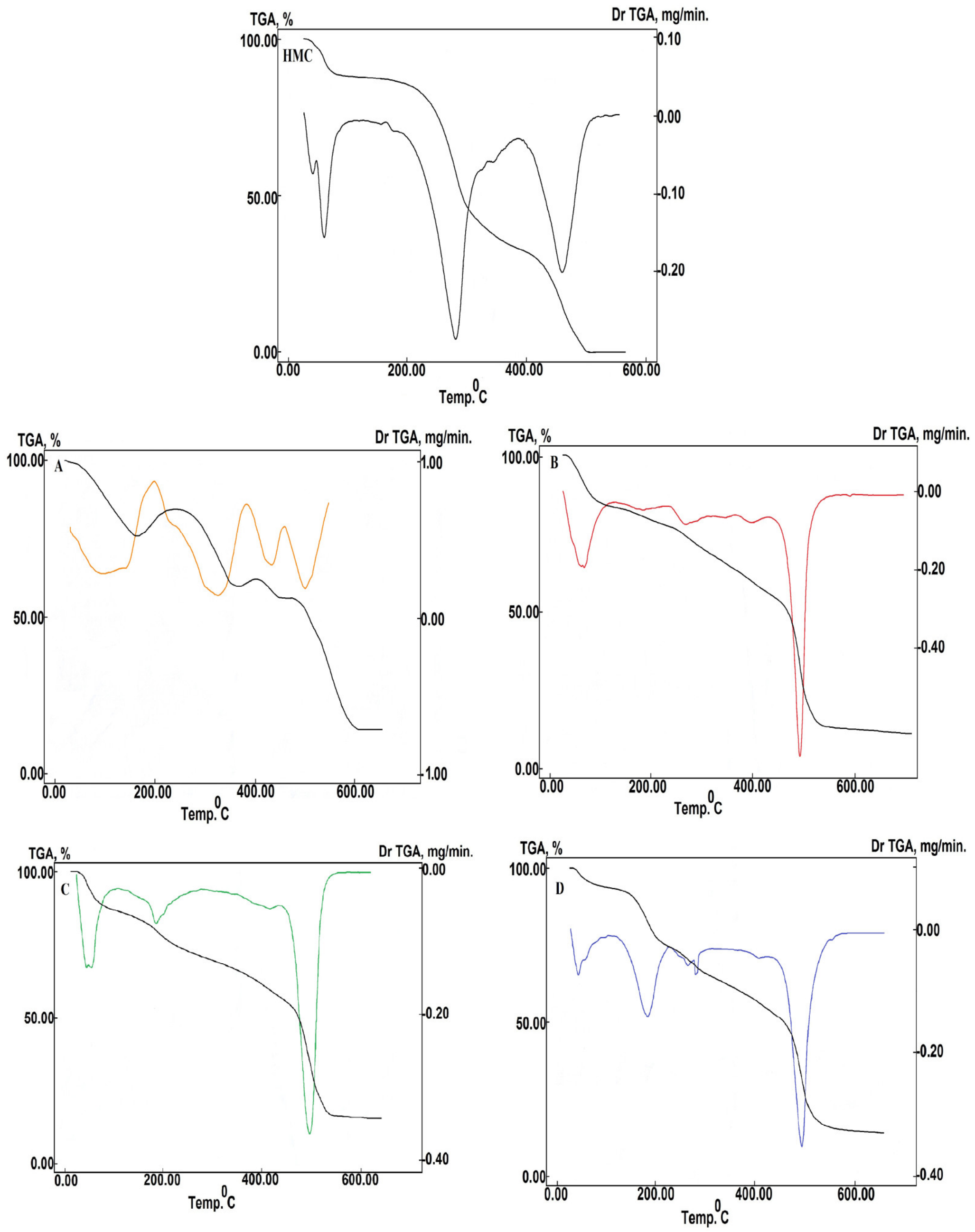

Figure S4. TG and DTG diagrams for CIP, HMC and their Zr(IV) complexes (**A**) $[ZrO(CIP)(HMC)(H_2O)Cl]Cl.5H_2O$, **(B)** $[ZrO(CIP)(HMC)(DMF)(H_2O)]Cl_2.10H_2O$, **(C)** $[ZrO(CIP)(HMC)(Py)(H_2O)]Cl_2.10H_2O$ and **(D)** $[ZrO(CIP)(HMC)(Et_3N)(H_2O)]Cl_2.3H_2O$.

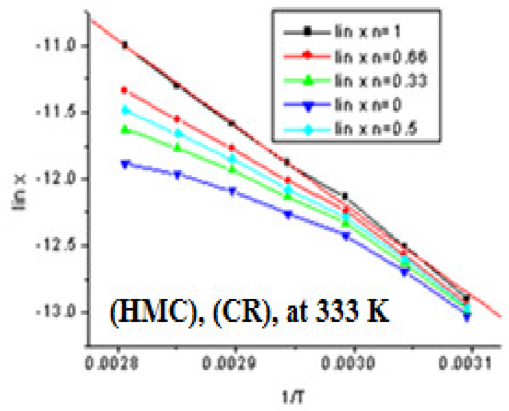
lin x n= 1
lin x n=0.66
lin x n=0.33
lin x n=0
lin x n=0.5
lin x
1/T
(HMC), (CR), at 333 K

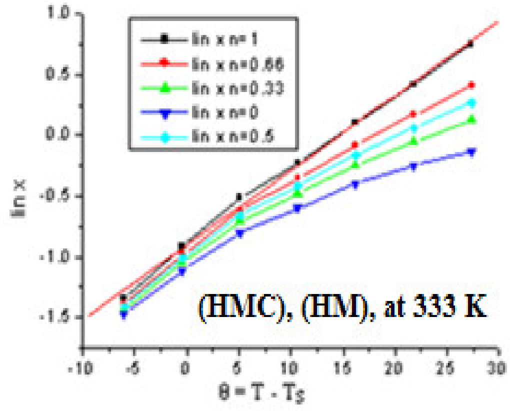
lin x n= 1
lin x n=0.66
lin x n=0.33
lin x n=0
lin x n=0.5
lin x
θ = T - T_S
(HMC), (HM), at 333 K

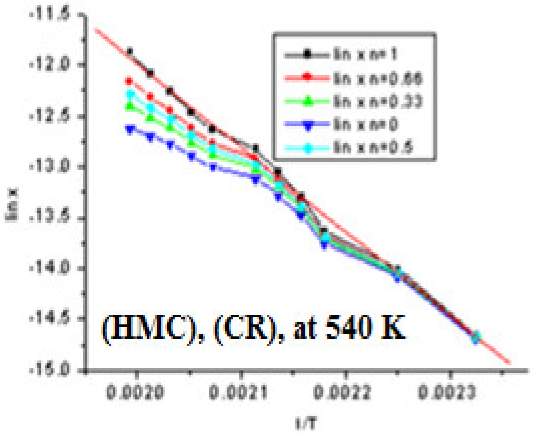
lin x n= 1
lin x n=0.66
lin x n=0.33
lin x n=0
lin x n=0.5
lin x
1/T
(HMC), (CR), at 540 K

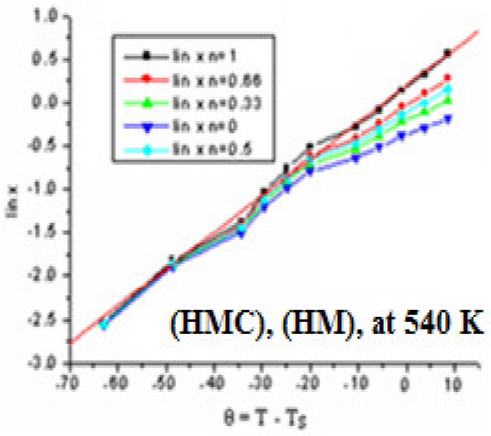
lin x n= 1
lin x n=0.66
lin x n=0.33
lin x n=0
lin x n=0.5
lin x
θ = T - T_S
(HMC), (HM), at 540 K

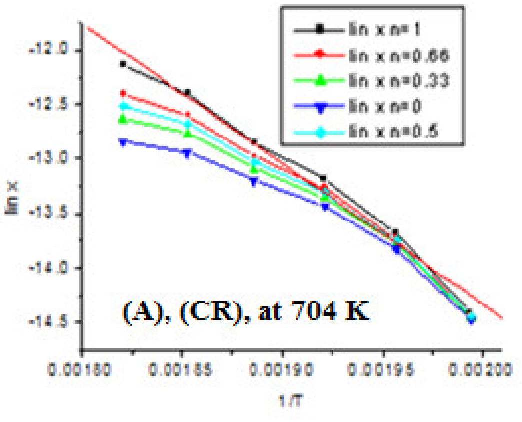
lin x n= 1
lin x n=0.66
lin x n=0.33
lin x n=0
lin x n=0.5
lin x
1/T
(A), (CR), at 704 K

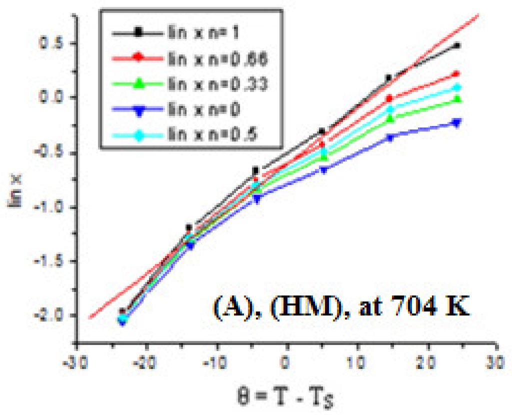
lin x n= 1
lin x n=0.66
lin x n=0.33
lin x n=0
lin x n=0.5
lin x
θ = T - T_S
(A), (HM), at 704 K

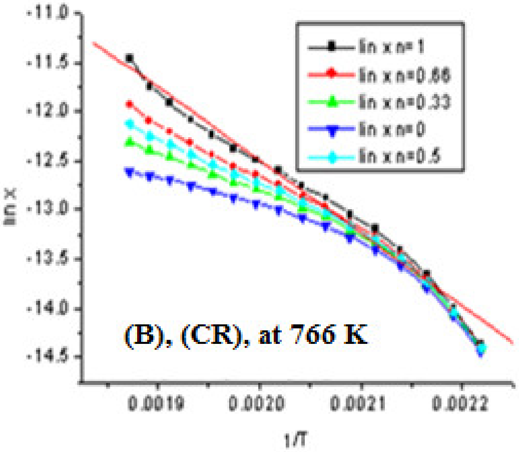
lin x n=1
lin x n=0.66
lin x n=0.33
lin x n=0
lin x n=0.5
(B), (CR), at 766 K
lin x
1/T

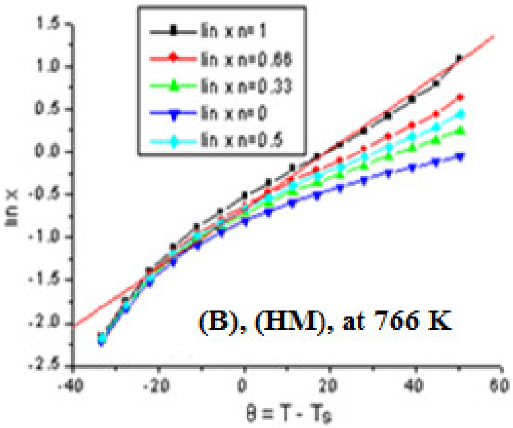
lin x n=1
lin x n=0.66
lin x n=0.33
lin x n=0
lin x n=0.5
(B), (HM), at 766 K
lin x
θ = T - Ts

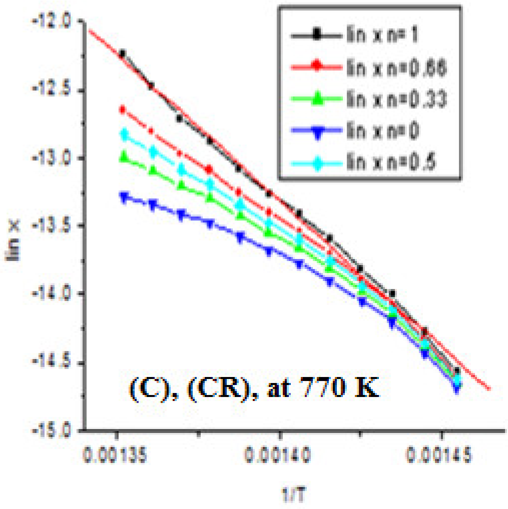
lin x n=1
lin x n=0.66
lin x n=0.33
lin x n=0
lin x n=0.5
(C), (CR), at 770 K
lin x
1/T

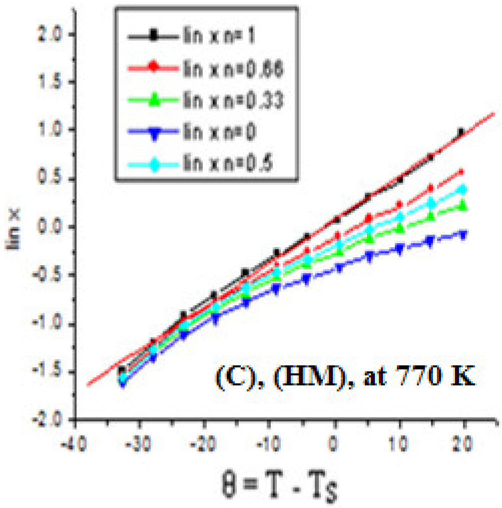
lin x n=1
lin x n=0.66
lin x n=0.33
lin x n=0
lin x n=0.5
(C), (HM), at 770 K
lin x
θ = T - Ts

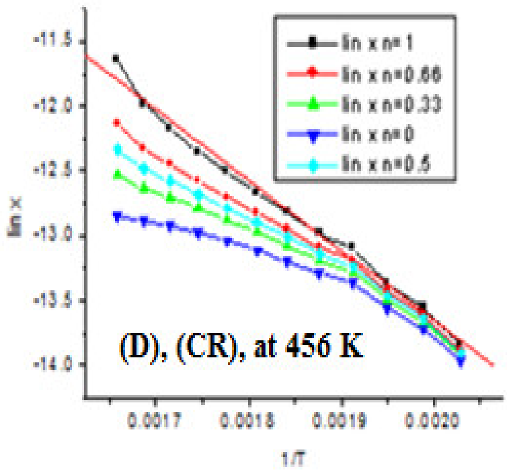
lin x n=1
lin x n=0.66
lin x n=0.33
lin x n=0
lin x n=0.5
(D), (CR), at 456 K
lin x
1/T

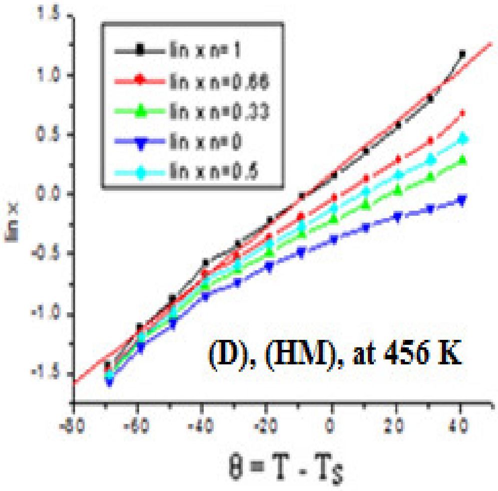
lin x n=1
lin x n=0.66
lin x n=0.33
lin x n=0
lin x n=0.5
(D), (HM), at 456 K
lin x
θ = T - Ts

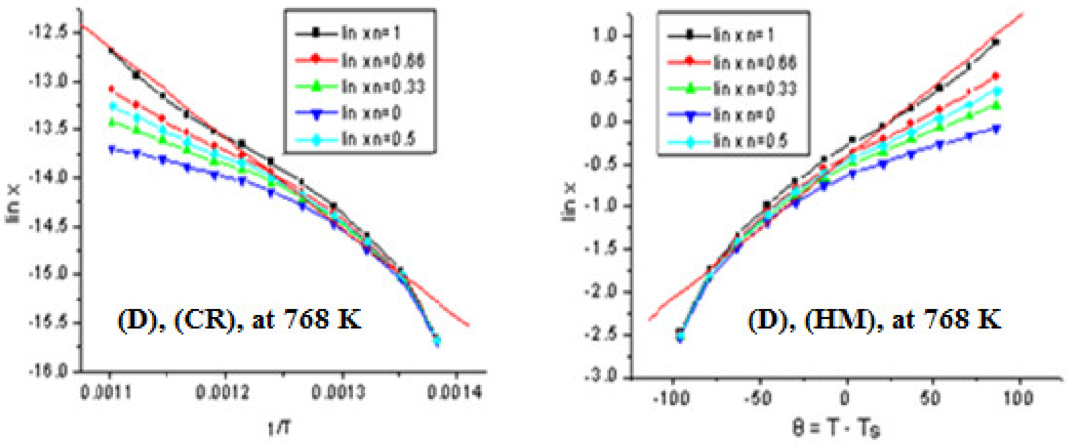

Figure S5. The diagrams of kinetic parameters for CIP, HMC and their Zr(IV) (**A)** [ZrO(CIP)(HMC)(H_2O)Cl]Cl.5H_2O, **(B)** [ZrO(CIP)(HMC)(DMF)(H_2O)]Cl_2.10H_2O, **(C)** [ZrO(CIP)(HMC)(Py)(H_2O)]Cl_2.10H_2O and **(D)** [ZrO(CIP)(HMC)(Et_3N)(H_2O)]Cl_2.3H_2O.

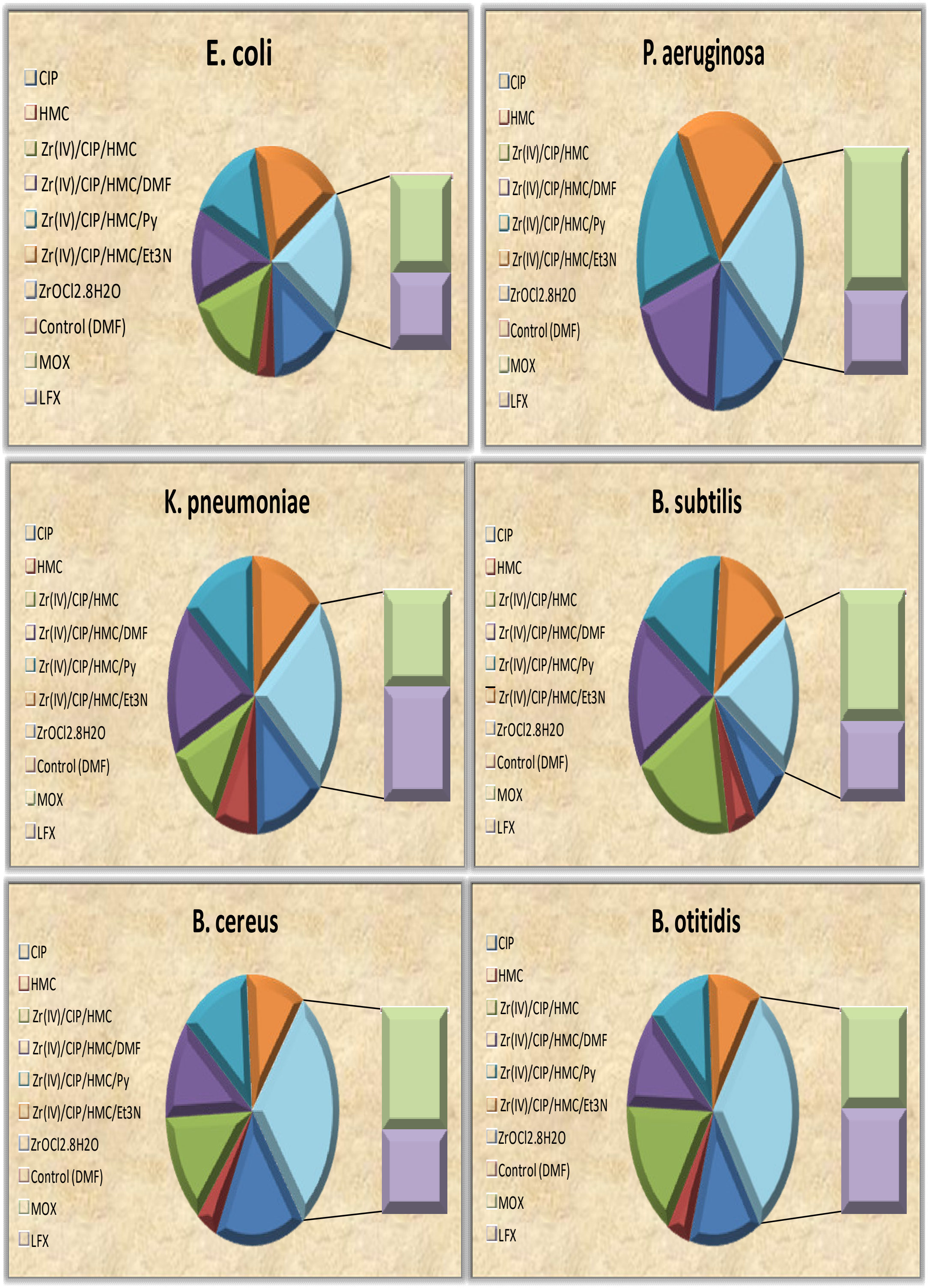

Figure S6. Antimicrobial activity of CIP, HMC and their Zr(IV) complexes.
